# The Relationship Between Stigma and Health-Related Quality of Life in People Living with HIV Who Have Full Access to Antiretroviral Treatment: An Assessment of Earnshaw and Chaudoir’s HIV Stigma Framework Using Empirical Data

**DOI:** 10.1007/s10461-018-2041-5

**Published:** 2018-02-07

**Authors:** Maria Reinius, Maria Wiklander, Lena Wettergren, Veronica Svedhem, Lars E. Eriksson

**Affiliations:** 1 Department of Learning, Informatics, Management and Ethics, Karolinska Institutet, 171 77 Stockholm, Sweden; 2 Department of Neurobiology, Care Sciences and Society, Karolinska Institutet, 141 83 Huddinge, Sweden; 3 Department of Infectious Diseases, Karolinska University Hospital, 141 86 Stockholm, Sweden; 4 Unit of Infectious Diseases, Department of Medicine, Karolinska Institutet, 171 77 Stockholm, Sweden; 5 School of Health Sciences, City, University of London, London, EC1V 0HB UK

**Keywords:** HIV stigma framework, HIV-related stigma, Health-related quality of life, Adherence to ART

## Abstract

The aim was to empirically test the tenets of Earnshaw and Chaudoir’s HIV stigma framework and its potential covariates for persons living with HIV in Sweden. Partial least squares structural equation modelling was used on survey data from 173 persons living with HIV in Sweden. Experiencing stigma was reported to a higher extent by younger persons and by women who had migrated to Sweden. As expected, anticipated stigma was related to lower *Physical functioning,* and internalized stigma to lower *Emotional wellbeing*. In contrast to that hypothesized by the HIV stigma framework, enacted stigma was not related to *Physical functioning* and no relationships were found between HIV-related stigma and antiretroviral adherence. These results indicate that the HIV stigma framework may need to be revised for contexts where a very high proportion of persons living with HIV are diagnosed and under efficient treatment.

## Resumen

El objetivo fue probar empíricamente los postulados del marco teórico del estigma del VIH (HSF) de Earnshaw y Chaudoir y sus covariables para personas con el VIH en Suecia. Se empleó el modelo de ecuaciones estructurales con la escala PLS (Partial least squares), sobre datos obtenidos en 173 encuestas a personas con el VIH en Suecia. El estigma experimentado fue más frecuente en jóvenes y mujeres emigrantes. Como se esperaba, el estigma anticipado estuvo asociado a bajo funcionamiento físico, y el internalizado a bajo bienestar emocional. En contra de las hipótesis del HSF, el estigma declarado no tuvo relación con el funcionamiento físico y no se encontró ninguna relación entre el estigma relacionado con el VIH y la adherencia a los antirretrovirales. Estos resultados sugieren que el marco teórico HSF debería ser ajustado para contextos en los que un alto porcentaje de las personas con el VIH están diagnosticadas y bajo tratamiento eficaz.

## Background

Feeling stigmatized due to living with HIV has been shown to be related to poor quality of life in different cultures [1–4]. Since the beginning of the HIV pandemic, experiencing stigma related to HIV has also been shown to be a barrier to treatment and prevention [5, 6]. Several theoretical frameworks have been presented for how HIV-related stigma manifests and operates [7–12]. One of the most cited frameworks, the HIV stigma framework by Earnshaw and Chaudoir [10], aims to capture the effects of HIV-related stigma on an individual level. The HIV stigma framework [10] covers mechanisms and outcomes of HIV-related stigma for individuals living with HIV and individuals who are (known or assumed to be) uninfected. The present study focuses on the part of the framework that addresses individuals living with HIV. According to the HIV stigma framework, the social phenomenon of HIV stigma impacts persons living with HIV through three different mechanisms: (1) Enacted stigma involving experiences of prejudice, marginalization and negative treatment by others due to one’s HIV, (2) Anticipated stigma involving expectations of enacted stigma and (3) Internalized stigma referring to when the stereotypes, labels and beliefs that constitute the stigma are endorsed and applied to oneself by the stigmatized person. The HIV stigma framework stresses the importance of differentiating between stigma mechanisms during measurement, since they may relate differently to health-related outcomes for persons living with HIV. When the framework was first described, the three stigma mechanisms were hypothesized to be related differently to psychological, behavioral and health outcomes for persons living with HIV [10]. In Earnshaw et al.’s [13] empirical evaluations of the HIV stigma framework for persons living with HIV, these outcomes of HIV-related stigma were specified as physical, behavioral and affective health and wellbeing. It has been suggested that enacted and anticipated stigma affects the individual’s physical health and wellbeing, since enacted and anticipated stigma can be a stressful experience, a stress considered so severe that physical health may be affected [13–15]. Anticipated stigma was thought to have consequences for behavioral health and wellbeing, since the individual may, for example, avoid medical care visits and skip medicine doses for fear of disclosing their HIV-status [5]. Internalized stigma was also proposed to influence behavioral health in the form of adherence to therapy due to feelings of not deserving treatment for their HIV infection or not deserving to feel well [5]. Internalized stigma could result in the individual having negative feelings about him or herself, e.g. feeling “less than” others, and has been associated with affective consequences such as mental health problems, for example depression [16]. It was therefore hypothesized that internalized stigma impacts affective health and wellbeing [13].

Isolated parts of the framework have been further explored [6, 17–22]. Anticipated stigma has been shown to be related to physical health in the form of more HIV symptoms among people living with HIV in the US, and social support has been found to buffer this relationship [17]. An extensive review of the literature has linked HIV-related stigma to behavioral health in the form of low antiretroviral adherence [6]. More specifically, internalized stigma has been shown to be related to lower antiretroviral adherence among people living with HIV in a US context [18] and also to be related to affective health in the form of depression among men who have sex with men in China [19]. Furthermore, the relationship between internalized stigma and behavioral health (low antiretroviral adherence) has been shown to be mediated by social support and depressive symptoms [21]. Internalized stigma has also been found to be related to engagement in care (mediated by HIV disclosure) and to higher virus levels for persons living with HIV in Italy [22]. The HIV stigma framework has also been expanded into a model where enacted and internalized stigma is related to perceived community stigma [20]. Perceived community stigma was found to be related to lower self-esteem (affective health) and lower antiretroviral adherence (behavioral health), relationships that were mediated by internalized stigma [20]. However, the original HIV stigma framework for persons living with HIV has, to our knowledge, only been evaluated empirically once, where empirical support for all the hypothesized relationships were found, except for the relationship between anticipated stigma and adherence [13].

According to Deacon [7] theories should constantly be reassessed in relation to empirical evidence. Since isolated parts of the framework have only been tested in a limited amount of studies [6, 17–22] and the entire original framework has only been tested once, and then only in a US context with a high rate of persons with HIV symptoms [13] (indicating suboptimal access to efficient treatment), we conclude that there is a lack of knowledge regarding the validity of the framework for different populations as well as in contexts with high access to contemporary antiretroviral treatment. Furthermore, since the stigma mechanisms are correlated [13], we argue that it is of significance to test all hypothesized relationships in the framework simultaneously. The present work is therefore an attempt to contribute to the theory building regarding factors that correlate with HIV-related stigma for individuals living with HIV. Our hypothesis was that the framework is valid for persons living with HIV in contexts other than those previously tested, including a context with very high access to antiretroviral therapy. This hypothesis was tested empirically with data collected from persons living with HIV in Sweden, where antiretroviral treatment is available to all, free of charge and where all UNAIDS/WHO 90-90-90 goals are met [23]. The aim of the present work was to test the tenets of the HIV stigma framework and its potential covariates for persons living with HIV in Sweden.

## Methods

### Procedure and Respondents

Data for this study was drawn from a cross-sectional survey of persons living with HIV recruited from the Department of Infectious Diseases at the Karolinska University Hospital in Stockholm, Sweden. The recruitment process for the data collection is described in detail elsewhere [24]. Inclusion criteria were (1) aged 18 years or older, (2) not being on their first appointment at the clinic and (3) being able to understand and complete the questionnaire in either Swedish, English or with assistance from a professional translator. Eligible respondents were included consecutively after signing informed consent. The study was approved by the Regional Ethical Review Board of Stockholm, Sweden (2008/1:12 with amendment 2013/335-32). Out of 360 potential respondents, 193 (54%) agreed to participate and returned a questionnaire. Of these, 132 returned a fully completed questionnaire (minimum response rate 37%) and a further 41 completed enough items for subscale scores to be calculated for all variables included in the path model. Responses from a total of 173 participants (48%) therefore constituted the sample in this study.

### Operationalization of Concepts in the HIV Stigma Framework

**Fig. 1:**
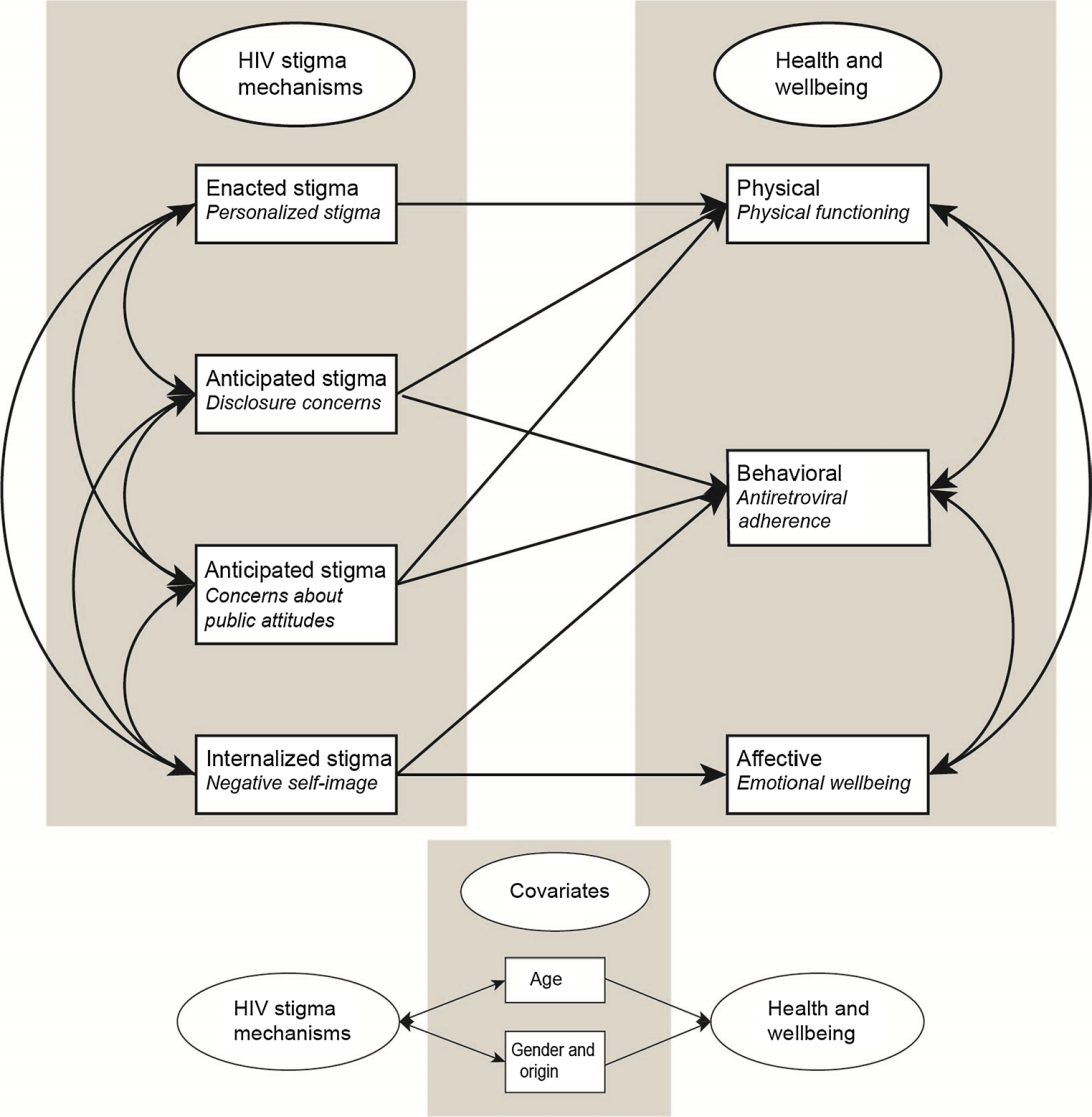
A hypothesized path model of the relationships between HIV stigma mechanisms and measures of health and wellbeing for persons living with HIV in Sweden based on Earnshaw and Chaudoir’s [10] HIV stigma framework, as further elaborated in Earnshaw et al. [13]. Age and a combined measure of gender and origin were included as potential covariates, hypothesized to be correlated to HIV stigma mechanisms and related to measures of health and wellbeing (presented schematically in this figure)

In the present study, a path model was specified based on the HIV stigma framework (Fig. 1). Alternative measures were used compared to when the model was tested by Earnshaw et al. [13]. The measures used in the present work are summarized in Table 1, with corresponding concepts in the HIV stigma framework. The measures used are described in detail in the Measures’ section below but in brief: enacted stigma was operationalized by *Personalized stigma,* anticipated stigma was operationalized by *Disclosure concerns* and *Concerns about public attitudes* and internalized stigma was operationalized by *Negative self*-*image* [25]. Physical health and wellbeing were assessed using self-reported physical functioning [26]. Behavioral health and wellbeing were measured by antiretroviral adherence, assessed through HIV viral load (VL) as a pseudo marker (HIV RNA < 50 copies/ml). Affective health and wellbeing were assessed using self-reported emotional wellbeing [26]. Age and a combined measure of gender and origin were included in the model as covariates to evaluate the accuracy of the framework for different groups.

**Table 1:** Concepts in the HIV stigma framework and corresponding measures used in the present study

Concept in the HIV stigma framework	Measure used in the present study (n items)	Brief description of measure used in the present study	Sample item	Cronbach’s alpha
Enacted stigma	Personalized stigma^a^ (16)	Perceived consequences of other people knowing about one’s HIV [25]	People have physically backed away from me when they learn I have HIV	0.957
Anticipated stigma	Disclosure concerns^a^ (8)	Concerns about disclosing one’s HIV status to others [25]	I worry that people may judge me when they learn I have HIV	0.876
	Concerns with public attitudes^a^ (7)	Concerns about other people’s opinion about HIV [25]	Since learning I have HIV, I worry about people discriminating against me	0.875
Internalized stigma	Negative self-image^a^ (8)	Feeling of being unclean, not as good as others because of HIV [25]	Having HIV in my body is disgusting to me	0.884
Physical health and wellbeing	Physical functioning^b^ (7)	To what extent one’s health interferes with ability to perform physical activities [26]	Is your health today good enough that you can do the following activities? Strenuous activities, e.g., heavy manual work, strenuous sports	0.904
Behavioral health and wellbeing	Antiretroviral adherence	Non-adherence as defined by one VL > 150 copies/ml or two or more consecutive VL > 50 copies/ml within the last 2 years of the condition with treatment ongoing at least 6 months prior to the evaluation period		
Affective health and wellbeing	Emotional wellbeing: negative effect^b^ (6)	Negative feelings during the last week [26]	I have felt sad	0.896
	Emotional wellbeing: positive effect^b^ (6)	Positive feelings during the last week [26]	I have felt liked	0.844

^a^Subscales of the HIV stigma scale [25]

^b^Subscales of the SwedQual [26]

### Measures

#### HIV Stigma Mechanisms

HIV stigma mechanisms were measured using the Swedish version of the HIV stigma scale [24] previously translated and adapted from the English HIV stigma scale [25]. The Swedish version of the HIV stigma scale is a 39-item scale that measures four dimensions of HIV-related stigma matching the three stigma mechanisms in the HIV stigma framework: the dimension *Personalized stigma* (16 items) measures enacted stigma, *Concerns about public attitudes* (7 items) and *Disclosure concerns* (8 items) measure anticipated stigma and *Negative self*-*image* (8 items) measures internalized stigma. All items are statements that the participant can agree or disagree with on a four-point Likert scale (1 = strongly disagree, 2 = disagree, 3 = agree, 4 = strongly agree). Scores from each item in a dimension were summarized to a subscale score, with higher scores reflecting more stigma [24]. Missing values were imputed where respondents had completed more than 50% of the items for a subscale using multiple Bayesian imputation [27] over five datasets in AMOS.

#### Physical Health and Wellbeing

The *Physical functioning* scale (7 items) from The Swedish Health-Related Quality of Life survey (Swed-Qual) [26], a Swedish health-related quality of life instrument, was used as a measure of physical health and wellbeing, i.e. the extent to which the health of the participant interferes with his or her ability to perform physical activities. Items were answered on a four-point Likert scale; the answer to each item was transformed into scores from 0-100. The mean score of the seven items belonging to the scale then constituted the *Physical functioning* scale score, with higher scores indicating better physical functioning [26].

#### Behavioral Health and Wellbeing

A measure of HIV VL was used as a pseudo marker of respondents’ adherence to antiretroviral treatment, which represents behavioral health and wellbeing. High concordance between adherence to antiretroviral treatment and VL has been shown for persons living with HIV in Sweden, with 94% of patients who reported optimal adherence having a VL < 50 HIV RNA copies/ml [28]. Respondents’ VL test results were obtained from the medical records via the National quality assurance registry InfCare HIV [28], which is an integrated part of the medical records, and were examined retrospectively for the previous 2 years. Respondents who had one VL > 150 copies/ml or two or more consecutive VL > 50 copies/ml within the past 2 years of having the condition, with treatment ongoing for at least 6 months prior to the evaluation period, were classified as non-adherent, all other respondents were considered as adherent.

#### Emotional Health and Wellbeing

Emotional health and wellbeing was measured with the subscales *Emotional wellbeing: negative effect* (6 items) and *Emotional wellbeing: positive effect* (6 items) from Swed-Qual [26]. Items were answered on a five-point Likert scale and scored in the same way as described for *Physical functioning* above. Higher scores indicate better emotional wellbeing for both scales. Lower scores for *Emotional wellbeing: negative effect* indicate that the respondent has felt nervous, tense, down, impatient, sad or annoyed during the past week whilst higher scores for *Emotional wellbeing: positive effect* indicate that the respondent has been a happy person who has felt liked, emotionally in harmony, and has had much to look forward to during the past week [26].

#### Sociodemographic and Clinical Variables

In the survey, respondents were also asked to report gender, age and country of birth. Information regarding years since diagnosis and route of HIV transmission for each participant was retrieved from the medical records via InfCare HIV [29]. Route of HIV transmission was classified into homo/bisexual, heterosexual, intravenous drug use or other.

### Statistical Analysis

Gender, age, country of birth and route of HIV transmission of the respondents were compared to non-respondents and to the total population of persons living with HIV in Sweden [29] to evaluate the validity of the sample used in the present study. Furthermore, gender, age and CD4 counts of the respondents were compared to published data from Earnshaw et al.’s [13] previous evaluation of the HIV stigma framework to evaluate the presence of any significant differences between the samples. T-tests were conducted for continuous and χ^2^-tests for categorical variables.

The hypothesized model of Earnshaw and Chaudoir’s [10] HIV stigma framework (Fig. 1) was tested using partial least squares structural equation modelling (PLS-SEM) with bootstrapping, in smartPLS3 [30]. Since missing data for the stigma scales were imputed with multiple imputations over five datasets, the bootstrapping PLS-SEM was replicated five times; estimates were then averaged. The soft modeling technique PLS-SEM was chosen due to the relatively small sample size together with different levels of data among the variables (binary, ordinal, continuous, with a majority of variables showing a non-normal distribution) [31]. Significance level was set to ≤ 0.05 (two tailed). Bootstrapping with 5000 replications in smartPLS3 was used to assess whether direct effects were significantly separated from zero (no significant change was chosen as method for dealing with significant changes during the bootstrap iterations) [31]. Significance for Pearson correlations coefficients were assessed in R statistics [32] with package Hmisc [33]. Standardized direct effects were interpreted as effect sizes according to guidelines for Cohens d, where values of 0.2–0.49 are interpreted as small, 0.5–0.79 as medium, and values exceeding 0.8 as large [34]. Effect sizes of correlations ranging between 0.1–0.29, 0.3–0.49 and > 0.5 were interpreted as small, medium and large respectively [34].

## Results

### Respondents

Participant characteristics are presented in Table 2. Of the 173 respondents, 43% were female and 51% were born in countries other than Sweden. Respondent ages ranged from 19 to 83 years and the mean age was 48.1 years (SD 11.4). The route of HIV transmission was heterosexual in 57% of the participants, homo/bisexual in 30%, intravenous drug use in 7% and other in 6%. The mean time since HIV diagnosis was 12 years (SD 8.0), 9% were classified as non-adherent and 4% had a CD4 count less than 200 × 10^6^ cells/ml. The characteristics of the sample (n = 173) were compared to the characteristics of the population of people living with HIV in Sweden and no statistically significant difference was found regarding gender (43% female vs. expected 38% (28), χ^2^ = 1.67, df 1, *p* = 0.20), path of transmission (57% heterosexual, 30% homo/bisexual, 7% intravenous drug use and 6% other vs. expected 50, 31 , 7 and 12% (28), χ^2^ = 6.53, df 3, *p* = 0.09) and adherence (9% non-adherent vs. expected 8% (28), χ^2^ = 0.366, df 1, *p* = 0.545). The sample had an underrepresentation of persons born in countries other than Sweden (51% vs. expected 59% (28), χ^2^ = 4.08, df 1, *p* < 0.05). Compared to eligible persons who declined to participate or who were excluded for other reasons, included participants had a statistically significant lower rate of persons born in countries other than Sweden (51% vs. expected 68%, χ^2^ = 21.95, *p* < 0.001) and a significantly lower rate of persons with a heterosexual route of HIV transmission (57% vs expected 70% (29), χ^2^ = 28.01, *p* < 0.001). Compared to the sample used by Earnshaw et al. [13] the Swedish sample had a lower rate of persons with CD4 counts below 200 × 10^6^ cells/ml (4% vs. 20% in Earnshaw et al. [13], χ^2^ = 27.52, *p* < 0.001).

**Table 2:** Sociodemographic and clinical characteristics of the sample of persons living with HIV in Sweden, n = 173

Characteristic	N (%)	Mean (SD)	Range
Age		48.1 (11.4)	19–83
Years since HIV diagnosis		12.0 (8.0)	0–29
Gender			
Female	74 (43)		
Male	99 (57)		
Country of birth			
Sweden	84 (49)		
Not Sweden	89 (51)		
Education			
Elementary school	32 (19)		
High school/secondary school	66 (38)		
College or university degree	63 (36)		
Other	12 (7)		
Route of transmission			
Heterosexual	99 (57)		
Homo/bisexual	51 (30)		
Intravenous drug use	12 (7)		
Other	11 (6)		
Non-adherent to antiretroviral treatment^a^	16 (9)		
CD4 count < 200 × 10^6^ cells/ml	7 (4)		
On antiretroviral treatment	165 (95%)		

^a^Non-adherence as defined by one VL > 150 copies/ml or two or more consecutive VL > 50 copies/ml within the last 2 years of the condition with treatment ongoing at least 6 months prior to the evaluation period

Descriptive statistics for the HIV stigma scale are presented in Table 3.

**Table 3:** Descriptive statistics of the HIV stigma scale results, for the sample of persons living with HIV in Sweden, n = 173

Sub scales	Range^a^	Mean score (SD)
Personalized stigma	16–64	35.0 (13.2)
Disclosure concerns	8–32	24.5 (6.0)
Concerns about public attitudes	7–28	18.5 (5.1)
Negative self-image	8–32	17.5 (6.5)

^a^Possible range of the scales and actual range of respondents’ answers are equivalent

### Testing the HIV Stigma Framework

#### Hypothesized Paths

**Fig. 2:**
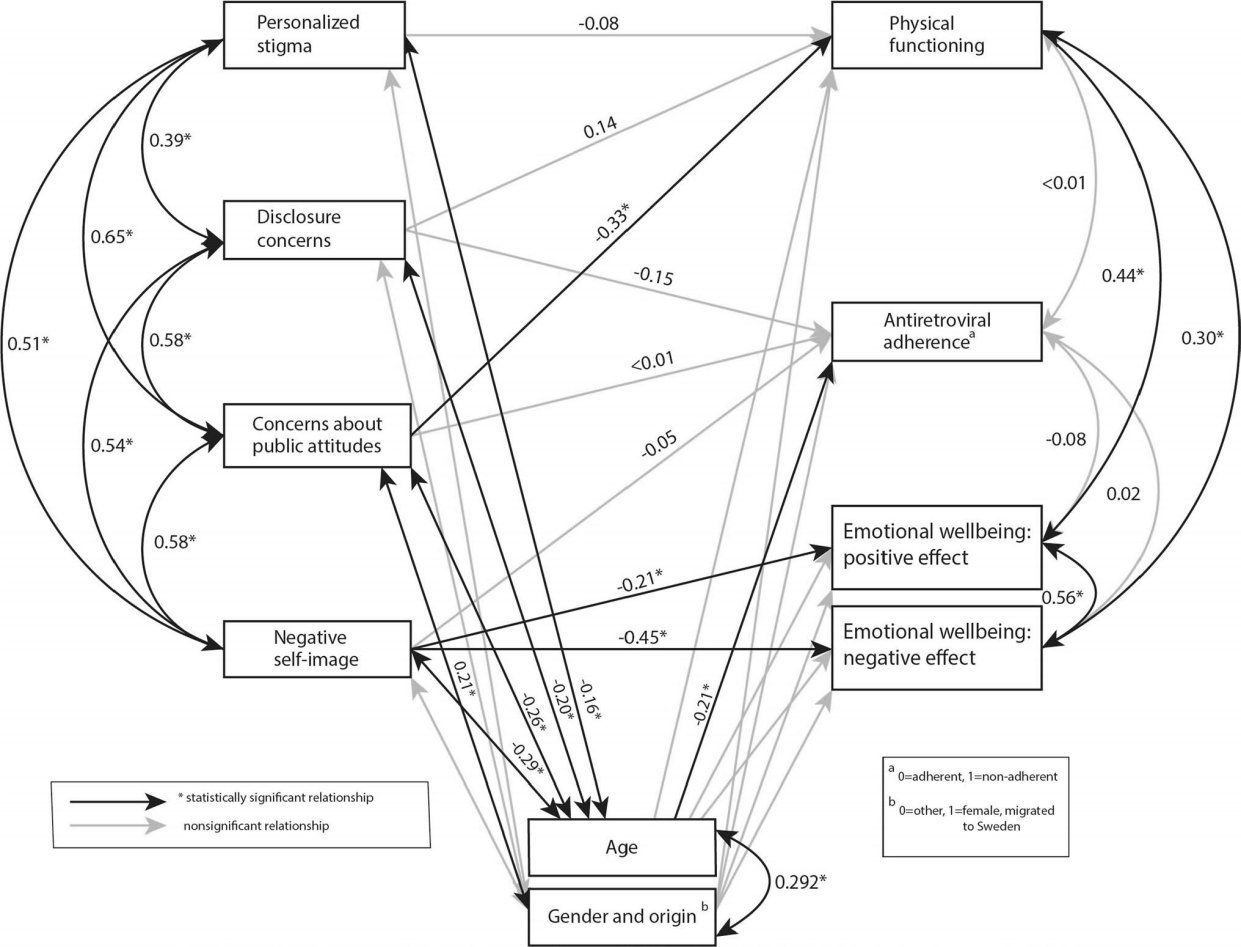
Results from the partial least squares structural equation modelling (PLS-SEM) analysis of the hypothesized path model presented in Fig. 1, empirically evaluated with self-reported and clinical data from persons living with HIV in Sweden. This figure illustrates the relationships with further data presented in Tables 4 and 5. The model depicts the relationships between measures of HIV stigma mechanisms (large boxes to the left), covariates (small boxes) and measures of health and wellbeing: Physical functioning, Antiretroviral adherence and Emotional wellbeing (boxes to the right). The estimates next to each arrow represent correlation coefficients and standardized direct effects. Black lines represent relationships that are statistically significant at a significance level of *p* < 0.05. Grey lines represent non-significant paths. Estimates for non-significant paths regarding covariates are not shown

Three out of eight hypothesized paths in the tested model had effects that were statistically significant, Fig. 2. Standardized direct effects are presented in Table 4. More anticipated stigma was associated with worse physical health and wellbeing (measured by *Physical functioning),* as hypothesized. However, this was only true for one of the two measures of anticipated stigma, *Concerns about public attitudes*, not for *Disclosure concerns*. As expected, Internalized stigma (measured by *Negative self*-*image*) was associated with lower affective health and wellbeing (measured by *Emotional wellbeing*, both *positive* and *negative effects)*. The effect sizes of these relationships were small. In contrast to that hypothesized in the model, Enacted stigma (measured by *Personalized stigma)* had no relationship to physical health and wellbeing (measured by *Physical functioning).* None of the HIV stigma mechanisms were related to behavioural health and wellbeing (measured by antiretroviral adherence). Regarding the hypothesized paths between covariates and measures of health and wellbeing, an inverse relationship was found between age and behavioural health and wellbeing (small effect size), indicating that persons that are non-adherent are of younger age. No other relationships were found between covariates and measures of health and wellbeing.

**Table 4:** Standardized direct effects for hypothesized paths in the HIV stigma framework for persons living with HIV (n = 173) calculated using partial least square structural equation modelling (PLS-SEM) with bootstrapping

Hypothesized path	Sample Mean	SD	*T* value	*p* value
Personalized stigma—physical functioning	− 0.08	0.11	0.77	0.47
Disclosure concerns—physical functioning	0.14	0.09	1.62	0.11
Concerns about public attitudes—physical functioning	− 0.33	0.11	3.07	< 0.05
Disclosure concerns—antiretroviral adherence^a^	− 0.15	0.11	1.11	0.31
Concerns about public attitudes—antiretroviral adherence^a^	< 0.01	0.12	0.16	0.87
Negative self-image—antiretroviral adherence^a^	− 0.05	0.11	0.49	0.63
Negative self-image—emotional wellbeing: positive effect	− 0.21	0.07	2.97	< 0.05
Negative self-image—emotional wellbeing: negative effect	− 0.45	0.07	6.90	< 0.05
Age—physical functioning	− 0.08	0.08	1.03	0.31
Age—antiretroviral adherence^a^	− 0.21	0.07	3.12	< 0.05
Age—emotional wellbeing: positive effect	− 0.03	0.06	0.53	0.60
Age—emotional wellbeing: negative effect	0.01	0.07	0.09	0.93
Gender and origin^b^—physical functioning	− 0.07	0.08	0.84	0.40
Gender and origin^b^—antiretroviral adherence^a^	0.12	0.09	1.28	0.20
Gender and origin^b^—emotional wellbeing: positive effect	0.09	0.07	1.46	0.15
Gender and origin^b^—emotional wellbeing: negative effect	0.06	0.07	0.89	0.37

^a^0, Adherent; 1, non-adherent

^b^0, other; 1, female, migrated to Sweden

#### Hypothesized Correlations

Correlation coefficients are presented in Table 5. In line with what is hypothesized by the HIV stigma framework, correlations were found between all HIV stigma mechanisms, and the scales from the Swed-Qual were also found to intercorrelate (Fig. 2, Table 4). Correlations between the HIV stigma scales were found to have medium to large effect sizes, and correlations between Swed-Qual scales small, medium and large effect sizes. Regarding the covariates included in the model, women born in countries other than Sweden had significantly more anticipated stigma (measured by *Concerns about public attitudes)* and inverse relationships were found between age and all measures of stigma. Effect sizes of these correlations were small.

**Table 5:** Pearson correlation coefficients between variables in the HIV stigma framework for persons living with HIV (n = 173)

Hypothesized path	Correlation coefficient	*p* value
Personalized stigma—disclosure concerns	0.39	< 0.05
Personalized stigma—concerns about public attitudes	0.65	< 0.05
Personalized stigma—negative self-image	0.51	< 0.05
Disclosure concerns—concerns about public attitudes	0.58	< 0.05
Disclosure concerns—negative self-image	0.54	< 0.05
Concerns about public attitudes—negative self-image	0.58	< 0.05
Personalized stigma—age	− 0.16	< 0.05
Personalized stigma—gender and origin	0.08	0.27
Disclosure concerns—age	− 0.20	< 0.05
Disclosure concerns—gender and origin	0.08	0.33
Concerns about public attitudes—age	− 0.26	< 0.05
Concerns about public attitudes—gender and origin	0.21	< 0.05
Negative self-image—age	− 0.29	< 0.05
Negative self-image—gender and origin	0.11	0.17
Age—gender and origin	− 0.30	< 0.05
Physical functioning—antiretroviral adherence	< 0.01	0.28
Physical functioning—emotional wellbeing: positive effect	0.44	< 0.05
Physical functioning—emotional wellbeing: negative effect	0.30	< 0.05
Antiretroviral adherence—emotional wellbeing: positive effect	− 0.08	0.79
Antiretroviral adherence—emotional wellbeing: negative effect	0.02	0.06
Emotional wellbeing: positive effect—emotional wellbeing: negative effect	0.56	< 0.05

## Discussion

In this article, Earnshaw and Chaudoir’s [10] HIV stigma framework for people living with HIV has been tested using data collected from persons living with HIV in Sweden, a context where all UNAIDS/WHO 90-90-90 goals are met [23]. The HIV stigma framework was only partly confirmed in this empirical test, indicating that the HIV stigma framework needs to be revised for contexts where a very high proportion of people living with HIV are diagnosed and under efficient treatment. As hypothesized in the model, anticipated stigma (measured by *Concerns about public attitudes)* was related to lower physical health and wellbeing. *Disclosure concerns,* also used as a measure of anticipated stigma, was not related to physical health and wellbeing, indicating that it is not *Disclosure concerns* themselves that are related to lower physical wellbeing, but *Concerns about other people’s negative opinions about HIV*. As also hypothesized, internalized stigma (measured by *Negative self*-*image)* was associated with lower affective health and wellbeing. Low behavioral health and wellbeing was only found to be inversely related to age and there was no significant relationship between enacted stigma (measured by *Personalized stigma)* and low physical health and wellbeing. There was a lower rate of persons with CD4 counts below 200 × 10^6^ cells/ml, i.e. there were more individuals whose HIV infection was in a better state, in the sample used in the present study than the sample used in the previous evaluation of the framework [13]. The HIV stigma framework, however, proposes to capture health-related effects of stigma for all persons living with HIV [10] and our hypothesis was therefore that the framework would also be valid for persons who are virally suppressed. This hypothesis could not be confirmed and a new hypothesis, drawn from our results, is that HIV-related stigma may have fewer or alternate outcomes for persons with good physical health and full access to antiretroviral treatment, such as the sample included in the present study. The impact that HIV-related stigma can have on the lives of persons living with HIV who have full access to antiretroviral treatment and high treatment adherence needs to be further explored in future research, which could lead to a revision of the HIV stigma framework for persons living with HIV.

The analysis of the covariates showed that women born in countries other than Sweden experienced anticipated stigma to a higher extent, and that younger age was related to higher levels of anticipated and internalized stigma. However, the effect size of these relationships was small. Earlier research shows contradictory results regarding whether the levels of HIV-related stigma differ with gender, origin and age. In a meta-analysis of health and demographic correlates to HIV-related stigma in a north-American context [2], only two of the articles included explored differences related to ethnicity, with contradictory results. In the same meta-analysis, differences related to gender were explored in three studies, with no statistically significant difference found. However, younger age was found to be related to higher levels of HIV-related stigma [2], in line with the results of the present study. Regarding the results of the present study, with a majority of respondents being under successful treatment, we want to emphasize that the effect of the covariates were small, indicating that persons experiencing high levels of stigma mechanisms may be found in all groups regardless of gender, origin and age.

Physical health and wellbeing (measured by *Physical functioning*) had, in contrast to earlier research, no relation to enacted stigma. To experience enacted stigma related to one’s HIV has earlier been shown to be a stressful experience that via neuroendocrine and sympathetic nervous system pathways impacts physical health [14, 15]. In the sample used in the present study, very few persons had CD4 levels under 200 × 10^6^ cells/ml or viral loads > 150 copies/ml. We therefore chose *Physical functioning* as a measure of physical health and wellbeing, since this variable had a larger variance, but this measure was not related to enacted stigma. Social support and community support have earlier been shown to buffer the association between anticipated stigma, stress and HIV symptoms, but not the association between enacted stigma, stress and HIV symptoms [17]. Further research is needed to explore if such factors have functioned as mediators and buffered the relationship between enacted stigma and physical health and wellbeing in a Swedish context. We did, however, find a statically significant relationship between physical health and wellbeing and anticipated stigma, which could possibly be explained by anticipated stigma being a stressful experience. Earlier research has also shown that people living with chronic illnesses who anticipate stigma were less likely to access care [35], which may also be a possible explanation to lower physical health and wellbeing for persons who anticipate stigma.

Behavioral health and wellbeing (as measured by VL as a marker of antiretroviral adherence) had no relation to HIV stigma mechanisms, which stands in contrast to earlier research where substantial empirical evidence has linked stigma to adherence difficulties [6, 18, 21] and less access to care [35], also in a Swedish context [36]. In Sweden, all persons diagnosed with HIV are obliged by the Swedish Communicable Diseases Act to avoid lost contacts with care and each individual is linked to a specialized HIV care center with quality assured care and treatment [23]; this may counteract negative effects of HIV stigma on behavioral health. There is also a possibility that respondents in the sample experience dimensions of stigma, e.g. layered stigma [37] not covered by the HIV stigma scale. Similarly, the hypothesized path between anticipated stigma and behavioral health and wellbeing was not confirmed when the HIV stigma framework was tested in an American context, and the authors related this to the cross-sectional design of the study [13]. The authors reasoned that anticipated stigma would have the strongest effect on future behavior, and therefore it would be preferable to measure this using a longitudinal design. We disagree with this reasoning and propose that anticipated stigma is something that can be a part of and affect everyday life. Therefore, a relationship between stigma and behavior should show even when investigated in a cross-sectional design, although causal relationships need to be investigated in longitudinal designs. One study has earlier shown that the relationship between HIV-related stigma and medical adherence was partially mediated by depression [38], something that is not addressed in the HIV stigma framework. However, if this mediated relationship was accurate in a Swedish context, we would have expected a stronger correlation between *Emotional wellbeing* and *Antiretroviral adherence*.

Affective health and wellbeing was, in line with earlier research, related to internalized stigma. Experiencing internalized stigma was associated both with having felt more negative feelings during the past week (negative affect) and having felt fewer positive feelings during the past week (positive affect). This corresponds to recent research where positive and negative affect were shown to mediate a relation between self-stigma and depression among Chinese men who have sex with men [19]. Future research should examine the relationship between internalized stigma, negative and positive affect, and depression in a population with high access to antiretroviral treatment.

Although the present study used a cross-sectional design and therefore does not provide information about causality among hypothesized relationships, we would like to address the question about causality in the HIV stigma framework. Earnshaw et al. [13] hypothesize causal relationships in the HIV stigma framework, where it is implied that higher stigma causes lower wellbeing. As shown, persons who anticipate stigma (measured by *Concerns about public attitudes)* to a higher extent rated lower *Physical functioning*. According to Earnshaw et al. [13], this may be explained as a causal relationship where low *Physical functioning* is caused by anticipated stigma among individuals. We propose that alternate explanations can be equally valid. A person with high *Physical functioning* could conceal their HIV status, which would prevent the individual from experiencing stigma [39], and low *Emotional wellbeing* could make a person more vulnerable to internalized stigma. We therefore propose a shift of focus from causal relationships to intertwined relationships between HIV-related stigma and measures of health and wellbeing.

### Implications for Care

When designing care for persons living with HIV it is valuable to know if individuals with certain background characteristics risk experiencing more stigma than others or are at higher risk for certain consequences of HIV-related stigma. Even if the patterns were not strong, the results of the present study imply that persons of younger age and women born in other countries than Sweden may be more exposed to HIV-related stigma. Therefore, in addition to broadly focusing stigma-reducing interventions, special resources targeting persons of lower age and women born in other countries than Sweden may be warranted. Furthermore, since the present study, along with earlier research, shows an inverse relationship between internalized stigma and emotional wellbeing, identifying and paying special attention to persons with internalized stigma may be warranted. Both the internalized stigma and the low emotional wellbeing could be targeted within a healthcare setting with support from a wide spectrum of healthcare professions. Existing cognitive and behavioral interventions that target internalized stigma have mainly been developed for women [40, 41], which is important, however there also seems to be a need for interventions targeting internalized stigma among men.

### Methodological Considerations

We did not use variables that were identical to those used when the framework was previously tested [13] and this may have affected the results. The areas in the HIV stigma framework that are hypothesized as being affected by HIV stigma (physical, behavioral and affective health and wellbeing) are, however, broad concepts and measures of health and wellbeing used in the present study match these concepts. In Earnshaw and Chaudoir’s [10] review of HIV stigma mechanism measurements, the HIV stigma scale [25] was considered to measure enacted, anticipated and internalized stigma. However, in their empirical test of the HIV stigma framework [13], an alternative measure for anticipated stigma was used with items more explicitly phrased about anticipation of enacted stigma in the future. In the present study, measures of disclosure concerns and concerns about public attitudes about HIV were used to measure anticipated stigma. These two scales include items both about what respondents anticipate happening in the future if their HIV status becomes known and what the respondent thinks the attitudes of people in general are regarding those with HIV, which are indicators of what would happen to the respondent if his or her HIV status became known. The inclusion of sociodemographic correlates into the path model rendered a large number of tested relationships, thus limiting the number of variables that could be included. Dropping the correlates could have given the opportunity to turn physical, behavioral and affective health and wellbeing into latent variables, measured by several manifest variables, but we prioritized the inclusion of sociodemographic correlates considering the lack of research concerning how HIV-related stigma varies across different sociodemographic backgrounds. Future research could examine if HIV-related stigma varies across persons with different paths of transmission. We decided against this since the number of persons whose route of transmission was intravenous drug use was low in this sample. Furthermore, the cross-sectional design precludes assumptions of causality among hypothesized relationships. The use of PLS-SEM supports reliability of the results, since PLS-SEM is preferred over covariance based SEM (CB-SEM) when data is non-normal and the model is complex [42]. In the model analyzed in the present study comprising 37 free parameters to estimate, a maximum of paths directed towards a construct was eight (including correlations as paths), for Personalized stigma. A minimum sample size would then preferably be ten times eight [42], i.e. a minimum sample size of 80, which is more than doubled in the present study. A diverse sample of respondents was included in this study, which we consider a strength, since studies that include self-reported data from persons living with HIV often use samples that do not reflect the population of people living with HIV. Although the rate of eligible respondents that declined to participate was high (45%), the sample was found to reflect the population characteristics of people living with HIV in Sweden, despite an underrepresentation of persons born in countries other than Sweden [24]. The persons attending the clinic from which the respondents were recruited live predominantly in a metropolitan area, which may have had an impact on the results. It is unknown if persons living with HIV experience stigma differently depending on where in the country they live. The prevalence of persons with problems with antiretroviral adherence is low in Sweden and in the analysis this subgroup is small. The results regarding antiretroviral nonadherence should therefore be interpreted with caution.

## Conclusion

Despite the limitations with regards to sample size presented, this study deepens the knowledge of how relations between stigma mechanisms and measures of health and wellbeing may differ depending on extent of access to care and treatment. The HIV stigma framework for persons living with HIV, that aims to capture effects of HIV-related stigma for all people living with HIV, was only partly confirmed in a Swedish context where all UNAIDS/WHO 90-90-90 goals are met [23]. The results in the present study indicate that the HIV stigma framework for persons living with HIV may need to be revised for contexts where a very high proportion of people living with HIV are diagnosed and under efficient treatment.
